# An Artificial Intelligence Supervision Program to Improve Video-Observed Therapy for Managing Tuberculosis Treatment: Development and Validation Study

**DOI:** 10.3390/tropicalmed11100272

**Published:** 2026-09-28

**Authors:** Xujun Guo, Junbo Bai, Xiang Wan, Howard Eugene Takiff, Yarui Yang, Changmiao Wang, Shan Huang, Chang Ma, Shengyuan Liu

**Affiliations:** 1 Department of Tuberculosis Control and Prevention, Shenzhen Nanshan Center for Chronic Disease Control, Shenzhen 518054, China; vanilla-canon@foxmail.com (X.G.); jfkddz@szns.gov.cn (J.B.);; 2 Shenzhen Research Institute of Big Data, The Chinese University of Hong Kong (Shenzhen), Shenzhen 518172, China; 3 Laboratorio de Genética Molecular, Centro de Microbiología y Biología Celular, Instituto Venezolano de Investigaciónes Científicas, Caracas 1020A, Venezuela

**Keywords:** tuberculosis, patient management, artificial intelligence, video-observed therapy, patient adherence

## Abstract

Although video-observed therapy (VOT) boasts substantial potential advantages in tuberculosis (TB) treatment management, insufficient staffing of community healthcare workers leads to inefficient and inadequate video review. The aim of the study was to develop an artificial intelligence (AI)-powered program to assist community healthcare workers by reviewing medication-taking videos, and then validate the program’s performance and effectiveness. We developed a video automatic review program (VARP) using a development dataset of 8000 medication-taking videos, which was partitioned at the participant level into 5781 videos for model training and 2219 videos for internal testing. We evaluated its performance versus community health workers based on TB specialists’ annotations and compared clinical management metrics before and after VARP adoption to verify its effectiveness. VARP combined YOLOv8n-based medication detection with dual-stream RGB and pose-based medication action recognition; the RGB stream used a 3D ResNet-50 backbone, whereas the skeleton stream used a modified 3D ResNet backbone (F_1_ = 0.90, 95% CI 0.89–0.91). In an independent post-deployment validation set of 7237 videos, VARP achieved significantly better accuracy (84.7%, 6133/7237 vs. 78.1%, 5653/7237) and specificity (72.7%, 1151/1584 vs. 0%, 0/1584) than community health workers (*p* < 0.001), with 75.3% (5449/7237) consistent judgments against TB specialists and vastly shorter video processing time (7.72 s vs. 23.12 h, *p* < 0.001). Compared with standard VOT management (n = 149), the VARP-supported group (n = 158) delivered faster daily monitoring completion (80.4%, 14,842/18,454 vs. 58.0%, 13,124/22,635 within 24 h, *p* < 0.001) and higher adverse event detection (67.7%, 107/158 vs. 35.6%, 53/149, *p* < 0.001). AI-powered review of TB medication videos optimizes key community VOT indicators such as review efficiency and assessment consistency, offering a practical, effective solution to address flaws in conventional manual VOT and strengthening routine TB monitoring.

## 1. Introduction

Tuberculosis (TB) is the leading cause of death from a single infectious agent [1]. The prolonged treatment for TB, currently a minimum of 4–6 months, often leads to patients dropping out during self-management of medication, which can result in treatment failure, drug resistance and on-going TB transmission [2,3]. Since the advent of anti-TB drugs in the 1940s, there has been a lack of effective models for managing adherence to the drug regimens, and as a result, the responsibility for adherence has usually been left to the TB patients.

In 1993, the World Health Organization (WHO) declared tuberculosis to be a world emergency [3]. Recognizing that effective treatment management is crucial for TB control, the WHO recommended directly observed therapy (DOT) for managing TB treatment. DOT requires that people with TB visit health facilities daily to take their medication under direct observation by healthcare providers. However, due to the high burden of time and the transportation costs associated with DOT, as well as the stigma imposed on patients making daily visits to the health facility, there have been many attempts to replace DOT with video-observed therapy (VOT). With VOT, the people on TB treatment use their mobile phones to record daily videos of themselves taking the medication and send the videos to healthcare providers for online review [4]. In mainland China, the standard first-line daily regimen is administered as fixed-dose combinations (FDCs), wherein isoniazid, rifampicin, pyrazinamide, and ethambutol are co-formulated into a single red tablet for the 2-month intensive phase, and isoniazid and rifampicin are co-formulated into a single black-orange capsule for the 4-month continuation phase. The readily distinguishable color of anti-TB medications has facilitated VOT implementation in China and promoted its adoption across multiple domestic regions.

Nanshan District in Shenzhen, China, is a highly economically developed administrative region [5]. Beginning in 2016, the Nanshan Center for Chronic Disease Control (NSCCDC) incorporated VOT into a TB patient management comprehensive system (TPMCS) and has achieved better treatment compliance and clinical results than are generally obtained with DOT [6]. However, the healthcare workers at the community health service centers (CHSCs) in Nanshan are responsible for up to 15 public health tasks in the national Basic Public Health Services program [7], and this heavy workload makes it impossible to carefully review the videos to determine whether the patients have actually taken their medication [8]. As a result, delays in reviewing the medication videos often exceed 24 h and all videos are generally judged as showing that the person took the medication, becoming classified as “medication proved”, leading to low confidence that the videos were carefully reviewed and that the designations are accurate. Additionally, with the rising awareness of privacy protection, TB patients are increasingly resistant to the intrusive collection of the personal information appearing in the long-term stored video recordings, leading to a persistently low rate of video uploads [9,10]. To encourage uploading of the daily videos, patients who upload videos of themselves taking at least 70% of the pills over the course of the 6 months of treatment received a monetary monthly reward. Unfortunately, however, this could lead to the uploading of deceptive videos so as to receive the reward without actually swallowing the medication, creating only the appearance of compliance [11].

To address these problems with VOT, we sought a management approach that reduces the burden of manual video review. It also eliminates long-term storage of private video data. Crucially, it must identify deceptive medication videos in which patients create the appearance of compliance without actually swallowing the medication. The growing application of artificial intelligence (AI) technologies for managing the treatment of chronic diseases provided possible solutions [12], particularly the analysis of medical videos with computer vision techniques that incorporate advances in deep learning convolutional neural networks (DCNNs) [13]. We incorporated this technology into a video automatic review program (VARP) that reviews medication videos uploaded by patients in real-time to assist healthcare workers in assessing adherence to standard medication-taking procedures. After verifying its performance, in October 2023 the program was integrated into the existing system for TB therapy management. This report describes the development of the VARP system, evaluates its performance, and assesses its effectiveness in the management of tuberculosis treatment.

## 2. Materials and Methods

### 2.1. VOT Procedures

After being diagnosed and prescribed a drug regimen at the NSCCDC, people with active, drug-sensitive pulmonary TB receive treatment management at a CHSC near their residence or workplace. Only patients on the standard FDC regimen are eligible for VOT; those requiring dose adjustments, additional separate tablets, or non-standard regimens are managed solely via DOT. Among all patients who meet the eligibility criteria for VOT management, each of them at the NSCCDC voluntarily choose to enroll in either VOT or DOT management, rather than being selected randomly, and monitoring begins concurrently with anti-TB treatment. Although both modalities are offered, no patient is managed under VOT and DOT concomitantly. People managed with VOT collect their medication at the CHSCs every 14 days and are instructed to upload daily medication-taking videos to the TPMCS server in real time via the VOT application on their mobile phones. The app also permits reporting any adverse reactions. Healthcare workers at the designated CHSCs are required to log into the system within 24 h after the video is uploaded and review the video to confirm that the patient has actually taken the prescribed medication. If patients fail to upload videos at the pre-set time or if healthcare workers reviewing the video cannot confirm that the medication was swallowed, the TPMCS sends an alert to the community healthcare workers, prompting them to telephone the patient to inquire about the missing videos, confirm that the medication was ingested or the reasons the medication was not taken. Patients without valid reasons for not uploading a video are advised to comply with VOT requirements by uploading the daily videos on time. Valid problems, such as adverse reactions, are addressed by the healthcare worker. Should the patients on a course of treatment fail to upload videos for more than 3 consecutive days, they are interviewed and usually switched to DOT management. The people on TB medication visit the NSCCDC for routine blood and urine tests and chest X-rays every 2 weeks for the two months of intensive drug therapy and then every month for the remaining four months of continuation therapy. Mycobacteria growth indicator tube (MGIT) cultures are obtained at the end of the 2nd, 5th and 6th months to monitor the effectiveness of the treatment, and patients with positive sputum cultures at the end of 2nd month will receive an additional sputum culture at the end of the 3rd month. The treatment is considered successful if the full course of therapy is completed with consecutive negative cultures during the final two months of treatment. NSCCDC TB specialists are responsible for training both the people being treated and the community healthcare workers in VOT management procedures.

### 2.2. Design of VARP

#### 2.2.1. System Integrated

The TPMCS on which the VARP is installed includes both mobile app- and web-based platforms. People on TB treatment use only the app interface, whereas healthcare workers and TB specialists at the NSCCDC have access to both the app and web platforms. Through this system, the people on a course of treatment upload medication-taking videos, report adverse drug reactions, communicate with healthcare workers and receive reminders about uploading daily videos and when to come to the health center to renew their supply of medication. Healthcare workers review and verify medication videos, document their determinations of medication adherence, provide health education, collect adverse reaction reports and record key follow-up details obtained from phone calls. TB specialists can monitor real-time medication adherence, track trends in adverse reactions and also calculate and allocate medication supplies across CHSCs.

#### 2.2.2. Development Dataset and Data Partitioning

The repository of medication-taking videos on NSCCDC’s server contains approximately 300,000 videos, along with the medication adherence classifications determined by healthcare workers. Using the random function in MySQL (Version 8.4.5), 8000 videos were selected from this repository and then reviewed and annotated by TB specialists at NSCCDC to constitute the model development dataset. To reduce participant-level information leakage, the development dataset was partitioned at the participant level using an approximately 3:1 ratio, with all videos from the same participant assigned exclusively to one subset. This resulted in 5781 videos for model training and 2219 videos for internal testing. All videos in the development set were required to show the patients performing medication-taking actions independently, without other people or moving objects in the footage.

Patients on VOT procedures were instructed to demonstrate three actions in sequence: medication display, placing all of the medication in the mouth, and swallowing. A video was classified as “medication proved” only when all three actions were clearly detected. Videos missing any of these actions were labeled “medication unproved”. Each training video was independently annotated by two senior TB specialists. Discrepancies were resolved by discussion to reach a consensus annotation.

#### 2.2.3. Functional Specifications

The people on TB treatment use the app with their mobile phone cameras to record and upload their medication-taking in real-time, without the possibility of storing videos for later upload. There are no requirements regarding video duration, lighting conditions or resolution for the recorded videos. VARP reviews uploaded videos in real-time and provides determinations on medication adherence. For cases judged as “medication proved”, only the adherence determination information is recorded and stored on the server and the original video is deleted. For cases judged as “medication unproved”, the system sends an alert to a designated healthcare worker, who must then call the person to verify whether the medication was taken and, if not, determine whether adverse reactions or other issues were involved. Healthcare workers may also view the videos. After the follow-up call, the healthcare worker will document the patient’s reported adherence status in the system, noting the reasons for non-adherence, when applicable. The system automatically deletes the video after this process.

#### 2.2.4. Principle of Operation

As shown in Figure 1, VARP analyzes medication-taking videos through two complementary processing pathways: medication detection and medication-taking action recognition. The outputs of the two pathways are integrated at the decision level to obtain the final medication-taking result. For medication detection, YOLOv8n is applied to the video frames to identify medication at the frame level. The detected medications are categorized as tuberculosis drugs only, other medications only, or mixed medications. Tuberculosis drugs only and mixed medications are considered valid. For medication action recognition, we adopt the RGBPoseC3D network as the core recognition model, with 32 frames sampled from each video as input. RGBPoseC3D adopts a dual-stream feature extraction structure, consisting of an RGB visual stream and a human skeleton stream. Specifically, the RGB image stream employs a 3D ResNet-50 backbone, whereas the skeleton stream employs a modified 3D ResNet backbone with a reduced channel configuration and without the first residual stage. The detailed architecture of the two streams is summarized in Table 1.

Human keypoints are detected using HRNet-W32 under the COCO 17-keypoint definition. For each sampled frame, the detected 2D keypoints are converted into heatmaps using a Gaussian kernel. For the k-th keypoint, the heatmap value at spatial location (i, j) is defined as $J_{\mathrm{kij}}=\exp\left(-\left[{\left(i-x_{k}\right)}^{2}+{\left(j-y_{k}\right)}^{2}\right]/\left(2*\sigma^{2}\right)\right)*c_{k}$, where (x_k_, y_k_) and c_k_ denote the keypoint coordinate and confidence score, respectively, and σ controls the Gaussian variance. The heatmaps from T frames are stacked to form a 3D heatmap tensor of size K × T × H × W. In this study, K = 17, T = 32, and H = W = 56, resulting in an input tensor of 17 × 32 × 56 × 56 for the skeleton feature extraction stream.

After global average pooling, the RGB stream and skeleton stream produce 2048-dimensional and 512-dimensional feature vectors, respectively. Each feature is projected to a 256-dimensional representation through a fully connected layer followed by a nonlinear activation: $F_{\mathrm{image}}^{’}=\sigma \left(F_{\mathrm{image}}w_{\mathrm{image}}+b_{\mathrm{image}}\right)$ and $F_{\mathrm{pose}}^{’}=\sigma \left(F_{\mathrm{pose}}w_{\mathrm{pose}}+b_{\mathrm{pose}}\right)$. The two modalities are then adaptively fused as $F_{\mathrm{fuse}}=\alpha_{\mathrm{pose}}F_{\mathrm{pose}}^{’}+\alpha_{\mathrm{image}}F_{\mathrm{image}}^{’}$. The weighting coefficients are derived from the complementary modality, with $\alpha_{\mathrm{image}}=\mathrm{softmax}\left(F_{\mathrm{pose}}^{’}w+b\right)$ and $\alpha_{\mathrm{pose}}=\mathrm{softmax}\left(F_{\mathrm{image}}^{’}w+b\right)$, allowing the fusion module to encode cross-modal relationships.

The fused feature is passed to a multilayer perceptron classification head containing two fully connected layers with 64 and 2 neurons, respectively. The two-dimensional output represents the class probabilities for medication-action recognition, and the class with the higher probability is selected. As defined in Section 2.2.2, a video is classified as “medication proved” only when both this valid medication detection and complete action recognition are satisfied. All other cases are classified as “medication unproved”.

### 2.3. Performance of VARP

#### 2.3.1. Independent Post-Deployment Validation Dataset

For independent post-deployment validation, 7237 additional medication-taking videos were randomly selected from the video repository. These videos were not used for model training, internal model selection, or parameter tuning, and were required to show the person performing the medication-taking action independently with no other people or moving objects in the video. This cohort was used to evaluate the generalization performance of VARP under real-world post-deployment conditions.

#### 2.3.2. Medication Determination

Each of the randomly chosen videos in the validation dataset was viewed and independently annotated by 2 senior TB specialists to classify medication-taking behavior, as described above for the training set. TB specialists further categorized the videos into 8 classes based on the completion status of these actions:

I: All three actions missing.

II: Medication display only.

III: Placing the medication in the mouth only.

IV: Medication display + placing the medication in the mouth (missing swallowing).

V: Swallowing only.

VI: Medication display + swallowing (missing placing the medication in the mouth).

VII: Placing the medication in the mouth + swallowing (missing medication display).

VIII: All three actions completed.

Among these categories, only class VIII was considered positive and all other classes were considered negative. As VARP evaluates “medication display” separately but combines “placing the medication in the mouth” and “swallowing” into a single judgment, the videos can be relabeled into four simplified categories: “I + III + V”, “II + IV + VI”, “VII”, and “VIII”.

#### 2.3.3. Statistical Analysis

Statistical analysis was performed using SPSS 22.0. A 2-tailed z-test was used to compare the sensitivity, specificity, accuracy (recall), positive predictive value (PPV, precision), and negative predictive value (NPV) of classifications by VARP versus health workers. The Mann–Whitney U test was applied to compare the video review time between the 2 groups. A *p* value < 0.05 was considered statistically significant. Additionally, the *F*_1_-score was calculated to evaluate model performance, which can be interpreted as the harmonic mean of precision and recall. To account for potential within-patient clustering in the independent post-deployment validation set (7237 videos from 992 participants), we additionally performed a cluster-adjusted analysis using participant-level bootstrap resampling with 1000 iterations. In each bootstrap sample, participants were resampled with replacement, and all videos from a selected participant were retained as a single cluster. The performance metrics (accuracy, sensitivity, and specificity) were recalculated for each bootstrap sample, and the 2.5th and 97.5th percentiles of the bootstrap distribution were used as the cluster-adjusted 95% confidence intervals. The original video-level point estimates were retained, and the cluster-adjusted confidence intervals were reported alongside them.

### 2.4. Effectiveness of VARP-Based VOT

#### 2.4.1. Study Subjects

This study compared all people with TB registered at the NSCCDC who received VARP-based VOT management (December 2023–May 2025) with historical VOT management by healthcare workers before VARP was implemented (October 2022–June 2023). The inclusion criteria were patients who (1) received outpatient TB treatment at NSCCDC and were managed by its affiliated CHSC; (2) were newly diagnosed with drug-susceptible pulmonary TB; (3) were managed with VOT; and (4) provided informed consent for the use of their treatment-related data in this study. Exclusion criteria included (1) the inability to independently comply with VOT due to conditions requiring caregiver assistance (e.g., hearing impairment, intellectual disability, mental disorders, speech impairment, or severe illnesses affecting self-care); (2) technical barriers hindering the proper operation of the TPMCS or VARP (e.g., smartphone hardware malfunctions, outdated software); and (3) the lack of stable internet connectivity in the person’s medication-taking environment.

#### 2.4.2. Statistical Analysis

Statistical analysis was performed with SPSS 22.0 software, using chi-square tests and Fisher’s exact tests to compare the indicators of management standardization, medication adherence, and treatment outcomes between the traditional VOT group and VARP-based VOT group, respectively, before and after VARP implementation in the TPMCS in December 2023. Welch’s *t*-test was applied to compare the age, and Mann–Whitney U test was used to compare the video review completion time between the two groups. A *p*-value of less than 0.05 was considered statistically significant.

## 3. Results

### 3.1. Development of VARP

#### 3.1.1. Development Process

The Shenzhen Big Data Research Institute developed VARP using a development dataset comprising 8000 medication-taking videos uploaded by 1384 people with pulmonary TB managed by the NSCCDC with VOT. The dataset was partitioned at the participant level into 5781 videos for model training and 2219 videos for internal testing. The cohort consisted of 877 males (63.4%) and 507 females (36.6%), with an average age of 34.36 ± 13.10 years. Throughout the development process, TB specialists from NSCCDC met frequently with the institute’s technical engineers regarding functional requirements, design solutions, workflow pathways, technical specifications, and presentation formats.

The final VARP architecture consisted of YOLOv8n for medication detection and a dual-stream RGB/pose medication action recognition network. The RGB stream used a 3D ResNet-50 backbone, whereas the skeleton stream used a modified 3D ResNet backbone. The action-recognition classifier outputs a two-dimensional probability vector, and the class with the higher probability is selected as the action-recognition result. The final VARP decision is obtained by integrating the medication detection and medication-action recognition results, as detailed in Section 2.2.4 and Figure 1.

#### 3.1.2. Benchmark Comparison and Model Selection

To evaluate model selection, the medication detection and medication action recognition components were benchmarked separately on the internal test subset. For medication detection, as Table 2 shows, representative classification, segmentation, instance segmentation, and object detection models were compared. YOLOv8 achieved the highest overall accuracy (88.28%) and specificity (83.81%), while maintaining a sensitivity of 90.47%, and was therefore selected as the medication detection model.

For medication action recognition, Table 3 shows that TDN, SlowFast, I3D, ST-GCN, PoseC3D, and RGBPoseC3D were evaluated. RGBPoseC3D achieved the highest accuracy (86.39%) and specificity (84.23%). Although PoseC3D achieved a higher sensitivity (95.18%), its specificity was substantially lower (73.84%). Therefore, RGBPoseC3D was selected because it provided the best overall balance between accuracy, sensitivity, and specificity.

Based on these benchmark experiments, YOLOv8 and RGBPoseC3D were selected for medication detection and medication action recognition, respectively. Their integration yielded an overall medication-taking recognition accuracy of 89.07%, sensitivity of 87.26%, and specificity of 90.91% in the corresponding internal evaluation.

#### 3.1.3. Interface and Features

VARP operates as an independent system deployed on a VARP-server with technical specifications shown in Table 4. The TPMCS is compatible with both iOS and Android operating systems but is currently available only with a Mandarin interface.

In addition to the server hardware environment, we quantified the computational requirements of the main components of the inference pipeline. For medication action recognition, 32 frames are sampled from each video. The parameter counts and FLOPs of the major components are summarized in Table 5.

The workflow begins when the people being treated for TB upload medication videos to the TPMCS-server. The videos are then automatically transmitted to the VARP-server for real-time analysis, and the VARP-server returns the assessment results as string data to the TPMCS-server. If the video is judged as “medication proved”, this result is recorded in the database and the video is deleted. Videos judged as “medication unproved” trigger an alert to healthcare workers to telephone the patient to verify that they took the medication or determine why they did not take it, after which the video is deleted.

As shown in Multimedia Appendix A.1, the VARP operational procedures for the people taking the medication are exactly the same as with VOT without VARP. They can check their medication verification status directly through their medication record cards in the app. As illustrated in Multimedia Appendix A.2 and Multimedia Appendix A.3, the VARP system eliminates the need for community healthcare workers to manually review all uploaded videos. With VARP, the community health workers simply log in to see VARP’s assessment results and the alerts indicating those patients whose videos were judged as “medication unproved” and require follow-up calls.

### 3.2. Performance of VARP

#### 3.2.1. Test Data

The independent post-deployment validation set included 7237 medication-taking videos from 992 people with TB that were reviewed and classified by TB specialists. A total of 593 of these were males (59.8%) and 399 were females (40.2%), with an overall average age of 35.51 ± 13.75 years.

#### 3.2.2. Utility

Using the classification by TB specialists as the reference standard, the *F*_1_-score of VARP reached 0.90 (95%CI 0.89–0.91), indicating good performance. Table 6 shows a comparison of the performance of VARP and healthcare workers in assessing medication adherence from the independent validation set videos.

VARP demonstrated significantly higher accuracy (84.7%, 6133/7237 vs. 78.1%, 5653/7237) and specificity (72.7%, 1151/1584 vs. 0%, 0/1584; cluster-adjusted 95% CI for difference 69.5–75.9%) than healthcare workers (all *p* < 0.001). We further adjusted for patient-level clustering by participant-level bootstrap resampling (1000 iterations). The cluster-adjusted 95% CIs were slightly wider but the conclusions remained unchanged (VARP accuracy: 83.4–86.2%; sensitivity: 86.5–89.7%; specificity: 69.5–75.9%; healthcare worker accuracy: 75.8–80.4%).

#### 3.2.3. Misclassifications

Table 7 compares VARP classifications against TB specialist annotations. Agreement was reached on 75.3% of the videos (5449/7237); the remaining 24.7% (1788/7237) were discordant. The categories with high misclassification rates by VARP were: misclassifying “including all three actions” (VIII) as “missing placement action”(VI), “missing swallowing action “ (IV), or missing both (II) in 380 videos (5.3%, 380/7237); misclassifying “missing showing action”(VII) as “only placing the medication in the mouth” (III), “only swallowing” (V) or “ all three actions are missing” (I) in 345 videos (4.8%, 345/7237); and misclassifying “missing showing action”(VII) as “including all three actions” (VIII) in 285 videos (3.9%, 285/7237).

#### 3.2.4. Time Required

Because VARP can review videos when they are uploaded, the median total duration of its review took 7.72 (IQR 6.69–9.44) seconds. In contrast, the median time elapsed between when a video was uploaded and when it was reviewed by a healthcare worker was 23.12 (IQR 5.48–56.20) hours. The healthcare workers spent a median of 16.67 (IQR 12.50–25.24) seconds reviewing each video. The differences in total and review duration between the two groups were both statistically significant (*p* < 0.001).

### 3.3. Effectiveness of VARP-Based VOT

#### 3.3.1. Baseline Characteristics

We then compared the results of videos classified by VARP against traditional VOT, in which the videos were reviewed by healthcare workers. This study included 158 people with TB managed with VARP-based VOT and 149 patients who were managed with the traditional VOT before the implementation of VARP. As shown in Table 8, there were no statistically significant differences between the two groups in demographic or clinical characteristics at the time of diagnosis (*p* < 0.05).

#### 3.3.2. Effectiveness Evaluation

People on TB treatment in the VARP-based VOT group uploaded a total of 18,454 videos, while those in the traditional VOT group uploaded 22,635 videos. When compared with the traditional VOT group, the VARP-based VOT group demonstrated significant advantages in the standardization of TB treatment management, with statistically significant differences in the percentage of videos classified as “medication unproved” (28.6%, 5285/18,454 vs. 0%, 0/22,635) (*p* < 0.001), the average time from video upload until the videos were reviewed (0.26 min vs. 1933.72 min, *p* < 0.001), and the percentage of daily videos whose management was completed within 24 h of uploading (80.4%, 14,842/18,454 vs. 58.0%, 13,124/22,635) (*p* < 0.001) (Table 9).

There were no statistically significant differences between the VARP-based VOT group and the traditional VOT group for video upload rates, medication adherence rates, consecutive missed medication rates, sputum smear conversion rates or treatment success rates (Table 10). Surprisingly, however, a statistically higher percentage of people being treated for TB in the VARP-based VOT group reported adverse reactions (67.7%, 107/158 vs. 35.6%, 53/149) (*p* < 0.001).

## 4. Discussion

### 4.1. Principal Findings

Although health education and family support play important roles in tuberculosis medication management [14,15], heavy workloads among healthcare workers limit health education provision and the development of family support for patients [16], reducing care quality and resulting in clinical safety risks [17,18]. Against this backdrop, financial incentives are commonly adopted to boost treatment adherence [19]. In the VOT program of Nanshan District, Shenzhen, workforce shortages raise two major concerns: an elevated risk of deceptive medication-taking behaviors associated with financial incentives, and privacy leakage risk caused by long-term video storage stemming from inefficient video review. AI-powered digital technologies are urgently needed to streamline workflows and ease workforce burden [20]. We developed the VARP using DCNN technology to address these problems and then used a random selection of medication videos to compare the performance of VARP to that of community healthcare workers in reviewing the videos. VARP demonstrated significant advantages across multiple key metrics, fully showcasing its accuracy and efficiency in medication video review. After VARP was then embedded into the TPMCS, we subsequently evaluated VARP’s impact on the standardization of medication management by community healthcare workers, on treatment adherence and treatment outcomes.

FDC pills simplify the medication regimens for TB, yet patients still need to take 2–10 pills daily. As the 6-month treatment regimen progresses and clinical symptoms are alleviated, they often have difficulty adhering to the treatment regimen [21,22]. We therefore used economic incentives to sustain VOT participation and encourage consistent uploads. However, financial rewards can also motivate deceptive videos in which patients appear to take the medication that they do not actually ingest. Over-tasked healthcare workers overlooked this risk, treating a video upload as equivalent to a confirmed ingestion. Healthcare workers were also given compensation for high VOT adherence for the people on TB treatment in their care, which may have contributed to their assessments that all VOT videos showed that the medication was taken.

Human action recognition (HAR) has been applied to fields such as sports, healthcare, security, smart homes, and transportation [23,24]. Digital adherence technologies (DATs) have been widely used in tuberculosis treatment management [25], but to our knowledge, few AI-driven deep-learning solutions have been published, especially for the automated review of TB medication-taking VOT videos. Sekandi et al. reported the sole published deep-learning work targeting this exact use case [26], which serves as a small-sample proof-of-concept feasibility study. In their work, binary single-stream classification was implemented on pre-screened high-quality videos, and multiple backbone ablation experiments were completed to evaluate different visual feature extractors. Nevertheless, this work remained limited to video-level binary classification on curated, pre-filtered high-quality footage; it did not extend to gesture-level multi-class annotation, multimodal feature fusion incorporating human pose information, or evaluation on unfiltered real-world field-collected VOT uploads.

As tools for measuring medication adherence, DATs face significant issues of both the under-reporting and over-reporting of medication adherence [25]. Of these, over-reporting is more concerning because it can fail to identify poorly adherent patients who need more support to complete the anti-TB drug regimen [26]. Although numerous studies have reported the advantages of VOT over DOT in the management of tuberculosis treatment [27], few studies have focused on whether VOT can accurately reflect medication-taking behaviors. Unlike many AI tools designed to detect and diagnose disease [28,29,30], VARP was developed to identify deceptive pill-taking behaviors. Therefore, high specificity and accuracy, rather than sensitivity, were given priority. Only videos that fully demonstrate the complete medication-taking process are classified as “medication proven”, thereby reducing the likelihood of over-reporting adherence. Compared with other DATs such as digital pillboxes or self-report medication apps, VARP operates automatically on the system server, is not affected by the person’s network connection or engagement and is also less prone to under-reporting.

VARP’s medication action recognition process combines RGB appearance and skeletal information. Skeletal keypoints are closely related to human actions and provide complementary motion information for medication action recognition. In our internal benchmark experiments, RGBPoseC3D achieved the highest overall accuracy and specificity among the evaluated video- and skeleton-based action recognition methods, including TDN, SlowFast, I3D, ST-GCN, and PoseC3D. These findings support the use of multimodal RGB and pose information for medication action recognition.

In the independent validation set comparisons, VARP outperformed community healthcare workers across multiple key metrics. Most notably, its far higher specificity (72.7%, 1151/1584 vs. 0%, 0/1584) (*p* < 0.001) demonstrates a greater capacity to detect deceptive medication-taking events. Although VARP’s sensitivity is lower than that of healthcare workers (88.1%, 4982/5653 vs. 100.0%, 5653/5653) (*p* < 0.001), overall accuracy remains superior (84.7%, 6133/7237 vs. 78.1%, 5653/7237) (*p* < 0.001). VARP also achieves a qualitative leap in review speed, reducing the video review time from 23.12 (IQR 5.48–56.20) hours for healthcare workers to just 7.72 (IQR 6.69–9.44) seconds for VARP, showcasing greater efficiency and usability. Moreover, VARP is highly user-friendly, allowing both the patients taking the medication and the community health workers to review the history of VARP’s classification of the videos, without the need to perform any operations beyond those required in traditional VOT management.

Following real-world deployment, VARP substantially reduced the proportion of daily video managements that were not completed within 24 h (19.6%, 3612/18,454 vs. 42.0%, 9511/22,635) (*p* < 0.001), leading to corresponding improvements in work efficiency. The reminder alert function of TPMCS—triggered by VARP when a video is judged as “medication unproved”—may play a critical role. The reminder alert is displayed as an app notification on the phone’s lock screen, prompting community healthcare workers to conduct a telephone follow-up. The reminder alerts are activated only for those videos classified as “medication unproved” by VARP, so that the healthcare workers need only to attend to these videos without having to review the “medication proved” videos. This emphasizes the necessity and urgency of action on the negative videos, creating a “focus of attention” that motivates healthcare workers to address issues promptly.

Compared with traditional VOT management, VARP-based VOT management has established a more stringent medication review mechanism, which imposes normative constraints on the actions shown in the videos of patients taking the medication. The people taking the medication receive faster feedback after uploading videos, which may serve as positive reinforcement for their uploading behaviors. In addition, healthcare workers can more promptly and proactively assess medication adherence and, when necessary, implement corresponding clinical interventions which further enhance trust and confidence in treatment management. Over 70% of medication-taking videos are classified as “medication proved” by VARP, eliminating manual video inspection for these cases, reducing verification bias and human errors arising from high volumes of video reviews and easing the workload within the video-review component for community healthcare workers—a benefit anticipated for automated pre-screening in prior published work [26]. Additionally, the classification of up to 30% of videos as “medication unproved” has the benefit of increasing direct contact between patients for TB and their healthcare workers through the VARP stimulated follow-up calls. This likely explains why there were more reports of adverse reactions with VARP than with VOT (67.7%, 107/158 vs. 35.6%, 53/149) (*p* < 0.001). However, except for the higher reporting of adverse reactions, there were no significant differences between VOT and VARP in medication adherence and treatment outcomes. Although our small sample size may have insufficient statistical power to show slight differences, the lack of differences may also be because the people taking medication received a monthly compensation of approximately 90 yuan (~13$) for video upload data during the period of traditional VOT management. However, due to policy adjustments, this compensation was canceled during the VARP-based VOT management phase. Studies have confirmed that economic incentives have a significant impact on adherence [31], and it is conceivable that VARP-based VOT management could have shown better results if the compensation policy had continued unchanged.

The pilot study conducted by Sekandi et al. in Uganda shows that DCNNs could support medication adherence monitoring where TB caseloads are high and healthcare workers are scarce [26]. Although the *F*_1_-score of VARP developed in this study is slightly lower than was seen with the 3D ResNet model in the Ugandan study (0.90 vs. 0.92), its specificity demonstrates a clear advantage (72.7% vs. 54.6%). The importance of VARP’s development lies in its ability to identify potentially deceptive medication-taking behaviors. Moreover, the Ugandan study excluded videos with controversial review results from the training and testing sets, whereas our study included all randomly selected video samples, making it more representative of real-world applications [32]. Although Shenzhen is a much more economically developed environment than Uganda, the application of AI technology helped address the shortage of healthcare workers in TB treatment management in both contexts. This suggests that improving work efficiency is a universal need across diverse settings, and the development of digital technologies that revolutionize healthcare services can both improve patient care and optimize the work of healthcare personnel.

### 4.2. Limitations

Although urine tests and pill counts are currently used as reference standards to verify actual medication ingestion [25], VARP, as an auxiliary tool for VOT management rather than an independent DAT, is constrained by the inherent limitations of video-based data collection in VOT. The VARP developed in this study only classifies whether a video contains valid visual evidence of patients ingesting medication. Accordingly, expert manual video annotations used as the ground truth in this work cannot overcome the fundamental limitations of video to confirm physiological uptake, which is consistent with the reference standard used in the prior study developing VOT auxiliary tools [26].

VARP can identify most common deceptive medication-taking behaviors, yet some remain undetectable, such as spitting out the medication after apparent swallowing or failing to ingest the full displayed dose, which could result in under-medication. Detecting all sophisticated deception would impose unjustified technical and recording costs, undermining field feasibility. Because participants already depend on financial incentives to keep uploading and disengage readily under added friction, stricter capture demands risk driving under-reporting and concealing true non-adherence, ultimately eroding trust in digital adherence data [25]. We therefore prioritized operational pragmatism—trading complete detection for sustainable patient cooperation and trustworthy frontline use.

Videos that VARP flags as “medication unproved” still trigger a telephone callback for self-reported confirmation, which is then logged as the final adherence determination —leaving residual scope for deceptive behavior to be recorded as “medication proved”. The direct questioning, however, may impose a psychological friction such that deceptive behavior is more likely to be occasional than sustained.

Additionally, the confusion matrix analysis revealed that VARP may misjudge certain medication behaviors. Off-diagonal errors routed to “medication unproved” may inflate unnecessary follow-up workload and erode review efficiency, whereas those mislabeled as “medication proved” carry clinical risk by clearing non-adherent uploads undetected. This indicates that there is still room for improvement in VARP’s accuracy. NSCCDC has accordingly contracted the developer to retrain the model on enriched misclassified clips, targeting both failure modes in the next iteration.

While VARP obviated manual video inspection for 71.4% of uploaded videos, reducing burden for the video-review component, we did not quantify time consumed by telephone follow-up and documentation triggered by “medication-unproved” alerts. Therefore, we cannot draw firm conclusions regarding changes in community health workers’ overall daily workload. Future studies should incorporate staff time-measurement or qualitative surveys to comprehensively assess total workload.

Although VARP reduces the possibility of privacy leakage, as proved videos are deleted immediately, and unproved videos are deleted after healthcare worker verification, the leakage risk is not completely eliminated.

Our findings are limited to patients with drug-susceptible TB on standard FDC regimens formulated as red tablets or black-orange capsules in China. Because the drug-detection component of VARP was trained using visual features of these region-specific pill appearances, its performance would degrade substantially when encountering TB medications with distinct tablet shapes, colors, or capsule designs used in other geographic settings. Direct out-of-the-box deployment without model fine-tuning or retraining with local drug imagery is therefore not feasible, which severely restricts the generalizability of the VARP. Furthermore, we did not collect independent annotations from the two TB specialists before consensus discussion, so inter-rater agreement could not be calculated, representing a limitation in evaluating the reliability of our reference standard. Our study also lacks a comparative evaluation of community health workers’ satisfaction with the VARP system compared with the original VOT system, and thus does not comprehensively assess the usability of VARP. Finally, this study did not adopt a randomized clinical trial design but instead compared retrospective data on TB treatment management before and after the implementation of VARP.

## 5. Conclusions

Integrating the AI-powered video review tool with VOT systems effectively addresses shortages in the community health workforce and improves the standardization and efficiency of tuberculosis treatment management. Nevertheless, it is not a standalone substitute for VOT, and evidence is still lacking regarding its ability to enhance patients’ medication adherence or optimize ultimate treatment outcomes.

## Figures and Tables

**Figure 1:**
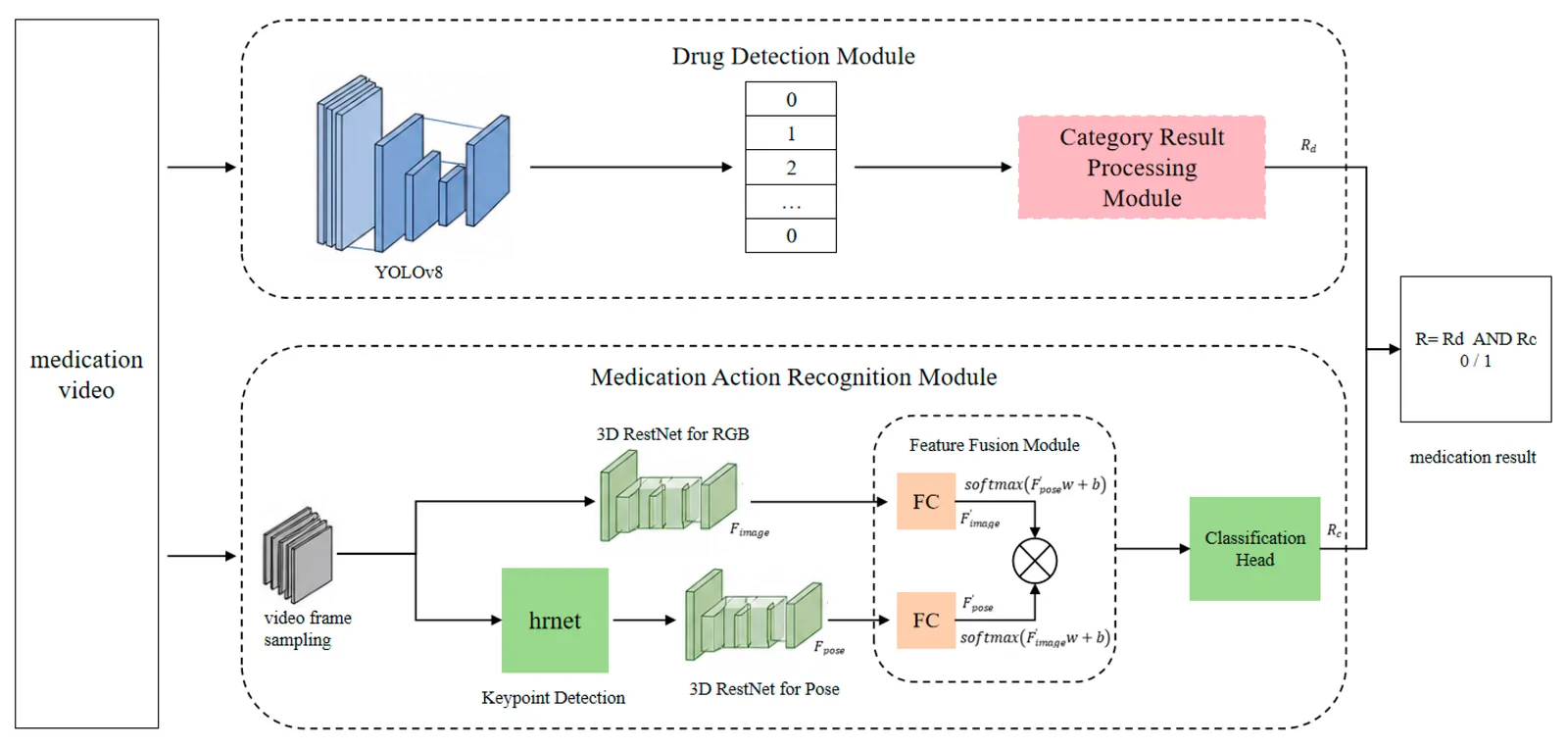
Working principle of VARP. VARP: video automatic review program.

**Table 1:** Architecture of the RGB and skeleton feature extraction modules.

Network Stage	RGB Image Feature Extraction	Skeleton Feature Extraction	Output
Input	3 × 32 × 224 × 224	17 × 32 × 56 × 56	—
Stem	Conv 1 × 7 × 7, 64; stride 1 × 2 × 2; MaxPool 1 × 3 × 3; stride 1 × 2 × 2	Conv 1 × 7 × 7, 32; stride 1 × 1 × 1	RGB: 64 × 32 × 56 × 56 Skeleton: 32 × 32 × 56 × 56
Residual stage 1	$\left[\begin{array}{c} 1\times 1\times 1,64\\ 1\times 3\times 3,64\\ 1\times 1\times 1,256 \end{array}\right]\times 3$	Omitted	RGB: 256 × 32 × 56 × 56 Skeleton: 32 × 32 × 56 × 56
Residual stage 2	$\left[\begin{array}{c} 1\times 1\times 1,128\\ 1\times 3\times 3,128\\ 1\times 1\times 1,512 \end{array}\right]\times 4$	$\left[\begin{array}{c} 1\times 1\times 1,32\\ 1\times 3\times 3,32\\ 1\times 1\times 1,128 \end{array}\right]\times 4$	RGB: 512 × 32 × 28 × 28 Skeleton: 128 × 32 × 28 × 28
Residual stage 3	$\left[\begin{array}{c} 3\times 1\times 1,256\\ 1\times 3\times 3,256\\ 1\times 1\times 1,1024 \end{array}\right]\times 6$	$\left[\begin{array}{c} 3\times 1\times 1,64\\ 1\times 3\times 3,64\\ 1\times 1\times 1,256 \end{array}\right]\times 6$	RGB: 1024 × 32 × 14 × 14 Skeleton: 256 × 32 × 14 × 14
Residual stage 4	$\left[\begin{array}{c} 3\times 1\times 1,512\\ 1\times 3\times 3,512\\ 1\times 1\times 1,2048 \end{array}\right]\times 3$	$\left[\begin{array}{c} 3\times 1\times 1,128\\ 1\times 3\times 3,128\\ 1\times 1\times 1,512 \end{array}\right]\times 3$	RGB: 2048 × 32 × 7 × 7 Skeleton: 512 × 32 × 7 × 7
Global average pooling	Yes	Yes	RGB: 2048 Skeleton: 512

**Table 2:** Benchmark comparison for medication detection.

Task Type	Model	Accuracy	Sensitivity	Specificity
Classification	ResNet50	84.18%	87.05%	78.20%
Classification	RegNet	85.31%	87.25%	81.34%
Segmentation	DDRNet	86.12%	88.12%	82.03%
Segmentation	HRNet	87.43%	89.53%	83.13%
Detection	Mask R-CNN	88.01%	90.60%	82.60%
Detection	YOLOv8	88.28%	90.47%	83.81%
Classification	ResNet50	84.18%	87.05%	78.20%

**Table 3:** Benchmark comparison for medication action recognition.

Model	Accuracy	Sensitivity	Specificity
TDN	81.63%	83.26%	79.96%
SlowFast	80.77%	82.12%	79.31%
I3D	82.34%	84.40%	80.15%
ST-GCN	79.05%	80.02%	78.02%
PoseC3D	84.81%	95.18%	73.84%
RGBPoseC3D	86.39%	88.43%	84.23%

**Table 4:** Working environment of VARP ^a^.

Configuration Item	Specific Parameter
CPU	Intel(R) Xeon(R) Silver 4216 CPU @ 2.10 GHz
Memory	128 GB
GPU	NVIDIA A100
OS	Ubuntu 20.04.6 LTS

^a^ VARP: video automatic review program.

**Table 5:** Computational complexity of the VARP inference pipeline.

Model	Parameters	FLOPs
YOLOv8n	3.2 M	8.7 GFLOPs/frame
HRNet-W32	28.59 M	490 GFLOPs
RGBPoseC3D	36.07 M	74 GFLOPs/clip
Total inference pipeline	67.9 M	approximately 600 GFLOPs/video

**Table 6:** Comparison of performance between VARP ^a^ and healthcare workers.

Metrics	VARP		Healthcare Workers		*p* Value
	% (n/N)	Cluster-Adjusted 95% CI ^b^ (%)	% (n/N)	Cluster-Adjusted 95% CI (%)	
Accuracy	84.7(6133/7237)	83.4–86.2	78.1 (5653/7237)	75.8–80.4	<0.001
Sensitivity	88.1(4982/5653)	86.5–89.7	100.0(5653/5653)	100.0–100.0	<0.001
Specificity	72.7(1151/1584)	69.5–75.9	0(0/1584)	0.0–0.0	<0.001

^a^ VARP: video automatic review program. ^b^ CI: confidence interval.

**Table 7:** Confusion matrix for classification of VARP ^a^ and TB ^b^ specialist.

TB Specialist	VARP			
	I + III + V	II + IV + VI	VII	VIII
I + III + V	61	38	14	13
II + IV + VI	12	136	19	135
VII	345	256	270	285
VIII	92	380	199	4982

^a^ VARP: video automatic review program. ^b^ TB: tuberculosis. I All three actions are missing. II Only showing the medication. III Only placing the medication in the mouth. IV Showing the medication and placing it in the mouth (missing swallowing action). V Only swallowing. VI Showing the medication and swallowing (missing placement action). VII Placing the medication in the mouth and swallowing (missing showing action). VIII Including all three actions.

**Table 8:** Demographic and clinical characteristics of people treated for TB ^a^ in the effectiveness study.

Metrics	VARP ^b^-Based VOT ^c^ Group (n = 158)	Traditional VOT Group (n = 149)	*p* Value
Gender, n (%)			0.72
Male	103 (65.2)	100 (67.1)	
Female	55 (35.8)	49 (32.9)	
Age (years), mean (SD ^d^)	45.54 ± 16.52	42.42 ± 15.15	0.12
Ethnicity, n (%)			0.74
Han	147 (93.0)	140 (94.0)	
Others	11 (7.0)	9 (6.0)	
Occupation, n (%)			0.42
Food and beverage industry	12 (7.6)	10 (6.7)	
Cadre staff	23 (14.6)	18 (12.1)	
Workers	19 (12.0)	24 (16.1)	
Domestic workers and unemployed	22 (13.9)	17 (11.4)	
Teachers	0 (0)	2 (1.3)	
Retired personnel	26 (16.5)	16 (10.7)	
Students	4 (2.5)	2 (1.3)	
Others	52 (32.9)	60 (40.3)	
Education level, n (%)			0.36
Junior high school and below	84 (53.2)	78 (52.3)	
High school	39 (24.7)	29 (19.5)	
Undergraduate and above	35 (22.2)	42 (28.2)	
Sputum smears or cultures at the time of diagnosis ^e^, n (%)			0.51
Positive	80 (50.6)	81 (54.4)	
Negative	78 (49.4)	68 (45.6)	
Cavity, n (%)			0.08
Yes	14 (8.9)	23 (15.4)	
No	144 (91.1)	126 (84.6)	
Registration classification, n (%)			0.13
People with no prior history of TB	148 (93.7)	145 (97.3)	
People with prior history of TB	10 (6.3)	4 (2.7)	

^a^ TB: tuberculosis; ^b^ VARP: video automatic review program.; ^c^ VOT: video-observed therapy.; ^d^ SD: standard deviation. ^e^ Either a positive sputum smear or culture is considered positive; otherwise, negative.

**Table 9:** Effectiveness for management standardization of healthcare workers.

Metrics	VARP ^a^-Based VOT ^b^ Group (n = 18,454)	Traditional VOT Group (n = 22,635)	*p* Value
Video review results, n (%)			<0.001
Medication proved	13,169 (71.4)	22,635 (100.0)	
Medication unproved	5285 (28.6)	0 (0)	
Video review completion time (min), median (IQR ^c^)	0.26 (0.22, 0.32)	1933.72 (1093.58, 3139.64)	<0.001
Time spent to complete daily management after a video is uploaded ^d^ (hours), n (%)			<0.001
<24	14,842 (80.4)	13,124 (58.0)	
≥24	3612 (19.6)	9511 (42.0)	

^a^ VARP: video automatic review program. ^b^ VOT: video-observed therapy. ^c^ IQR: interquartile range. ^d^ Healthcare workers need to log into the system, check the medication adherence results given by VARP for the VARP-based VOT group, or manually review the videos from the traditional VOT group. If the video is judged as “medication unproved”, healthcare workers must contact the person with TB who uploaded the video and document the result to complete daily management.

**Table 10:** Effectiveness for medication adherence and treatment outcomes of people treated for TB ^a^.

Metrics	VARP ^b^-Based VOT ^c^ Group (n = 158), n (%)	Traditional VOT Group (n = 149), n (%)	*p* Value
Fraction of the videos uploaded ^d^ (%)			0.57
<50	38 (24.1)	39 (26.2)	
50–69	25 (15.8)	22 (14.8)	
70–89	77 (48.7)	64 (43.0)	
≥90	18 (11.4)	24 (16.1)	
Fraction of the doses taken ^e^ (%)			0.15
<90	8 (5.1)	3 (2.0)	
≥90	150 (96.7)	146 (98.0)	
Number of times the medication was discontinued for ≥3 days ^f^			0.12
0	153 (96.8)	138 (92.6)	
1	4 (2.5)	5 (3.4)	
≥2	1 (0.6)	6 (4.0)	
Adverse events reported by people taking TB medication			<0.001
Yes	107 (67.7)	53 (35.6)	
No	51 (32.3)	96 (64.4)	
Treatment time required for clearance of bacilli from the sputum ^g^ (months)			0.10
2	33 (97.1)	34 (81.0)	
3	1 (2.9)	6 (14.3)	
5	0 (0)	2 (4.8)	
6	0 (0)	0 (0)	
Treatment success rate ^h^			0.70
Yes	152 (96.2)	142 (95.3)	
No	6 (3.8)	7 (4.7)	

^a^ TB: tuberculosis. ^b^ VARP: video automatic review program. ^c^ VOT: video-observed therapy. ^d^ The number of videos uploaded divided by the number of videos that should have been uploaded. ^e^ The number of medication doses taken divided by the number of doses that should have been taken according to the treatment regimen. The numerator includes doses confirmed through video as well as self-administered doses reported by the person taking TB medication during telephone follow-ups. ^f^ Instances when patients missed their doses for three or more consecutive days voluntarily, excluding cases when medication was suspended due to medical advice. ^g^ Only patients with a positive smear or culture at the start of therapy could be compared for clearance of the bacilli from the sputum, so the numbers of VARP-based VOT group and traditional VOT group are 34 and 42, respectively. Negative culture results are deemed as clearance of bacilli from the sputum. ^h^ A person treated for TB is considered to have achieved treatment success upon completing the full course of therapy with consecutive negative cultures during the final two months of treatment.

**Figure A1:**
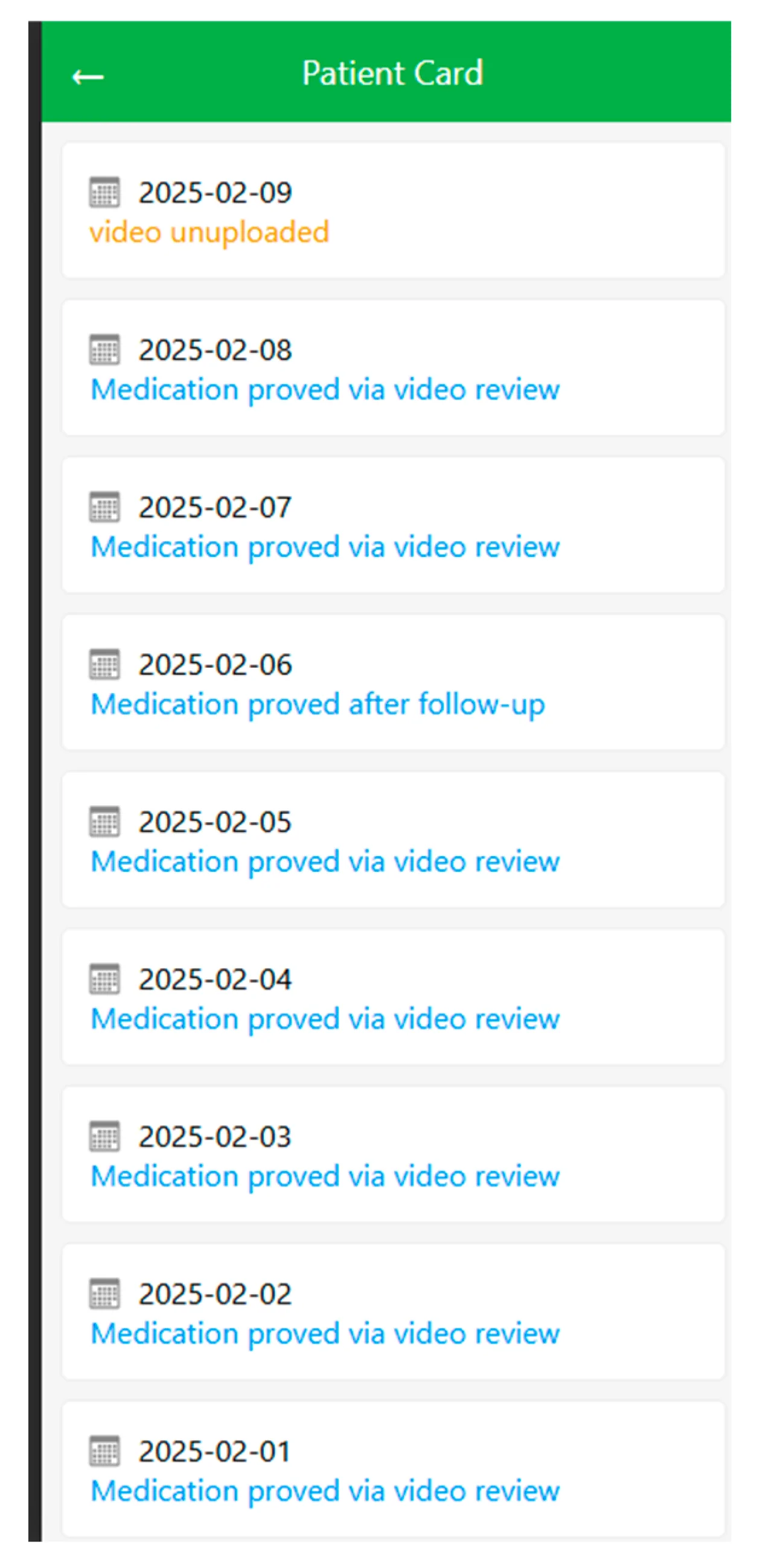
The medication record card interface of VARP results output.

**Figure A2:**
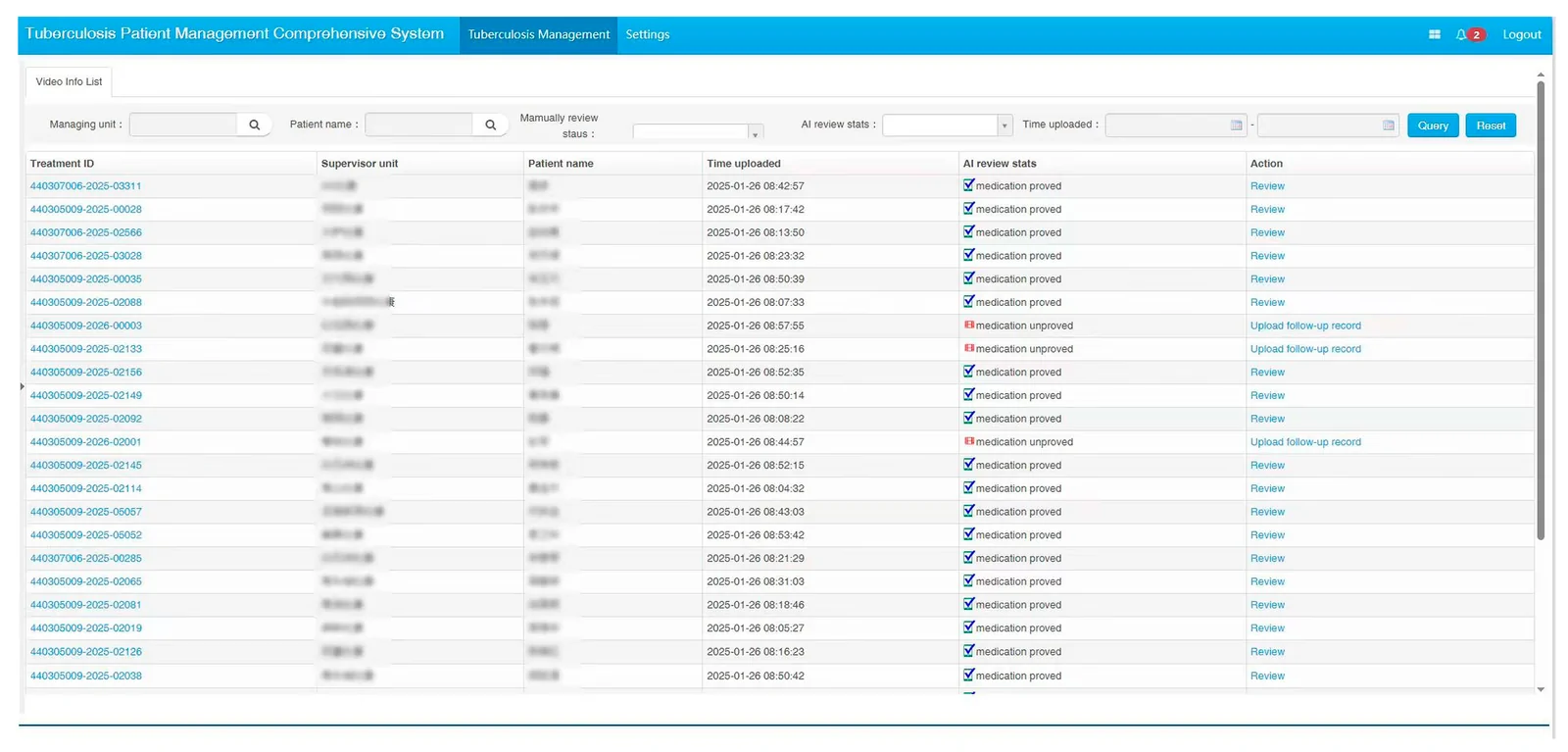
The community healthcare workers’ video management interface showing VARP video review results.

**Figure A3:**
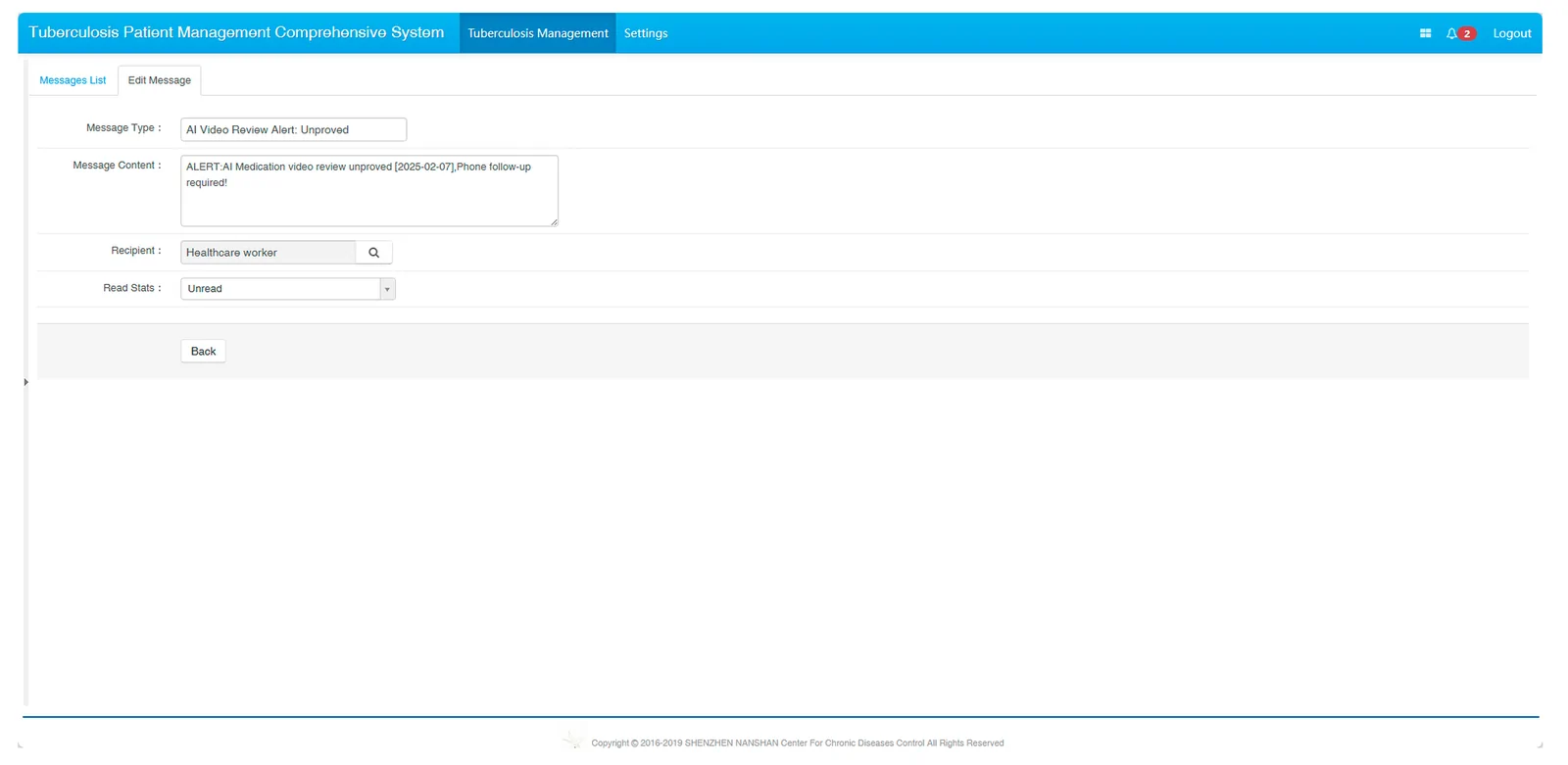
The community healthcare workers’ alert notification interface.

## Data Availability

The data analyzed during this study are not publicly available to protect participant confidentiality, as they cannot be fully de-identified. Researchers seeking further information regarding this study may contact the corresponding author.

## Abbreviations

The following abbreviations are used in this manuscript:

AI	Artificial intelligence
CHSCs	Community health service centers
CI	Confidence interval
DATs	Digital adherence technologies
DCNN	Deep convolutional neural network
DOT	Directly observed therapy
FDC	Fixed-dose combination
NSCCDC	Nanshan Center for Chronic Disease Control
ResNet	Residual convolutional neural network
TB	Tuberculosis
TPMCS	Tuberculosis Patients Management Comprehensive System
VARP	Video automatic review program
VOT	Video-observed therapy
WHO	World Health Organization
